# Visual detection of hemolysis in a blood bag before issue

**DOI:** 10.4103/0973-6247.76013

**Published:** 2011-01

**Authors:** N. Choudhury, Ankit Mathur

**Affiliations:** *Senior Consultant, Department of Transfusion Medicine; Tata Medical Center, Kolkata; India*; 1 *Consultant, Rotary Bangalore ttk Blood bank, Bangalore, India*

## Introduction

Hemolysis may occur in a red blood cell (RBC) unit during blood collection, transportation, preservation and or different stages of handling in the blood bank. Visual detection of hemolysis of a unit is possible usually by observing the color of the supernatant plasma. It is a good practice to observe the color of the plasma just before issue to avoid any inadvertent serious transfusion of a hemolysed blood unit. However, practically it is difficult once the stationary blood unit is disturbed or there is no time to centrifuge every unit of cross-matched blood to observe the plasma color.

## Observation

**Figure 1:**
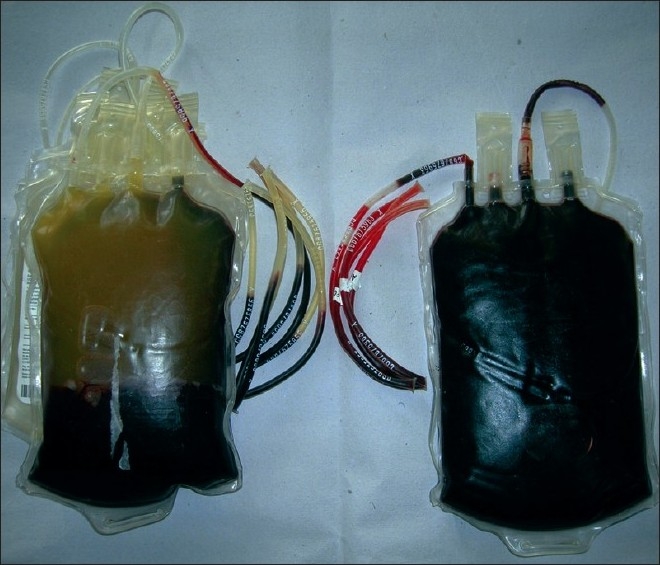
Colour of plasma in the segments

It is highly recommended to keep segments on the side of every blood unit for cross-match and further use. The plasma level of the blood unit may be disturbed during routine handling but the separated plasma level in the segment is not disturbed. When a blood unit is hemolysed due to any reason like thermal injury, blood in the bag and also the segment is lysed. Because the plasma is not disturbed in the segments, hemolysis of the unit can be easily observed just before issue. The illustration showed clear color differences between the plasma color in segments of hemolysed and normal blood units [Figure 1].

## Pathophysiology of the event

The elements of “red blood cell storage lesion” include: morphological changes, slowed metabolism with a decrease in the concentration of adenosine triphosphate (ATP), acidosis with a decrease in the concentration of 2,3-diphosphoglycerate (2,3-DPG), loss of function (usually transient) of cation pumps. There is consequent loss of intracellular potassium and accumulation of sodium within the cytoplasm, oxidative damage with changes to the structure of Band 3 and lipid peroxidation, and loss of parts of the membrane through vesiculation. Together, these events risk compromising the safety and efficacy of long-stored red blood cells, reducing their capacity to carry and release oxygen, promoting the release of potentially toxic intermediates like, free hemoglobin which act as a source of reactive oxygen species.[1,2]

Hemolysis during blood collection and storage is the most severe manifestation of the red blood cell (RBC) storage lesion. It represents either the rupture of RBCs with the release of hemoglobin (Hb) directly into the suspending fluid or the loss of membrane-bound Hb in microvesicles.[1]

## Preventive measure

To prevent these untoward consequences of hemolysis, regulatory agencies have set hemolysis standards as a condition for RBC storage system licensure. In the United States, this standard was historically a mean hemolysis of less than 1%, and in Europe, less than 0.8%.[1]

The actions which can be taken to reduce RBC lysis during storage:

1. The refrigeration of blood is expected to slow metabolism and thus enhance preservation. Thus maintaining temperature between 2-6 C reduces glucose metabolism and improve red cell viability.[3,4]
2. Ployvinyl chloride bags plastisized with DEHP are the standard red cell storage container. The presence of DEHP reduces hemolysis fourfold during storage by intercalating into red cell membrane.
3. Leukocyte reduction also improves red cell storage by removing highly metabolic and active blood component of white cell that would make the bag more acidic.
4. Additive solutions containing 30 mM of mannitol reduce hemolysis and further increase the osmolarity of the solution.[4]
5. A protective layer is recommended between RBC storage and freezing device to maintain the temperature in the RBC storage unit or transportation units. RBC should not be stored close to air-vent in the walk in coolers where temperature may drop lower than 1-6C.
6. RBC must be transported in an appropriate container that maintains the recommended temperature.
7. During component separation centrifugation speed higher than 5000 g induces hemolysis, which should be avoided.[5]
